# Clinical significance of PLT for diagnosis and treatment monitoring in imported malaria

**DOI:** 10.1038/s41598-024-66929-7

**Published:** 2024-07-09

**Authors:** Shui Fu, Qi-Lei Hu, Liang Zhang, Xiao-Jun Han

**Affiliations:** 1 Clinical Laboratory Department, First People’s Hospital of Linping District, Hangzhou, Hangzhou, Zhejiang Province People’s Republic of China; 2 Clinical Laboratory Department, The People’s Hospital of Cangnan Zhejiang, Wenzhou, Zhejiang Province People’s Republic of China

**Keywords:** Malaria, Platelet, Inflammation, Imported, Efficacy, Prognosis, Infectious diseases, Biomarkers

## Abstract

To evaluate the clinical significance of PLT, MPV, and PDW in monitoring malaria treatment efficacy and predicting disease progression. A total of 31 patients with imported malaria were selected as the observation group, while 31 non-malaria patients with fever were selected as controls. The observation group was subdivided into a complication group and a non-complication group according to the occurrence of complications during treatment. Additionally, on the 1st day (within 24 h), the 3rd day, and the 5th day following admission, a comprehensive blood routine examination, Plasmodium microscopic examination, and colloidal gold assay were conducted. The blood routine examination results were compared before and after treatment among patients in the observation group and the control group. Moreover, the study involved dynamic monitoring and analysis of the levels and variations in PLT, MPV, and PDW within both the complication group and the non-complication group. The Plasmodium density was negatively correlated with PLT before treatment. There were significant differences were observed in PLT, MPV, and PDW (*P* < 0.05) within the observation group before and after treatment. Notably, there were no significant alterations in red blood cell (RBC), hemoglobin (Hb), and white blood cell (WBC) counts (*P* > 0.05) within the observation group before and after treatment. The PLT, MPV, and PDW levels in the complication group and the non-complication group exhibited an upward trend after treatment. Further, the PLT of patients in the complication group was significantly lower than that in the non-complication group. Additionally, the PLT, MPV, and PDW levels in the complication group and the non-complication group increased gradually from the time of admission to the 3rd and 5th day of treatment. Notably, the PLT in the complication group was consistently lower than that in the non-complication group. The continuous monitoring of PLT, MPV, and PDW changes plays a crucial role in assessing malaria treatment efficacy and prognosis in these individuals.

## Introduction

Malaria is a parasitic disease caused by human exposure to Plasmodium, and it has evolved into a globally significant health concern. On June 30, 2021, the WHO announced that China had successfully achieved malaria elimination status. This achievement marked China as the first country in the Western Pacific Region to receive malaria-free certification from the WHO in the last three decades^1^. Despite such achievement, malaria elimination status does not imply the complete absence of malaria cases. With frequent international exchanges and cooperation, as well as increases in international travelers, imported malaria may pose a persistent global threat^2–4^. Malaria is characterized by rapid progression and a short course of disease. A delayed diagnosis can lead to missed opportunities for optimal treatment, and inaccurate efficacy monitoring can worsen a patient’s condition. Ultimately, patients may experience severe complications such as acute intravascular hemolysis, renal failure, and other life-threatening conditions, which seriously endanger the life of patients. At present, malaria should be detected through examinations such as blood routine and etiological examinations (peripheral blood smear microscopy, rapid Plasmodium antigen detection, and Plasmodium gene detection)^5^. Malaria diagnosis primarily relies on blood smear staining microscopic examinations, which is considered the gold standard. However, such method has limitations as it can be time-consuming and dependent on the expertise of healthcare providers. In countries where indigenous Plasmodium has been eliminated, malaria has become a less common disease with a lower prevalence. As such, most health providers conduct simulation learning based on theories and diagrams. The lack of practical experience among healthcare providers can result in inaccurate Plasmodium examination results under a microscope, thereby impacting the diagnosis and treatment of malaria patients^6^. Despite being known for its simplicity and speed, rapid Plasmodium antigen detection cannot be employed to monitor antimalarial treatment responses^7^. Plasmodium gene detection is a nucleic acid diagnosis method based on polymerase chain reaction (PCR). In recent years, there has been significant progress in macrogene detection technology, which can not only help identify different Plasmodium species but also detect genes related to drug resistance in Plasmodium parasites. Such detection methods feature high specificity and sensitivity, but there are difficulties in popularizing them as clinical routine methods in hospitals, especially in primary hospitals, due to the fewer kit types and higher requirements on hardware and software^8^. Therefore, there is an urgent need to explore common and efficient experimental indicators for the preliminary screening, efficacy monitoring, and prognosis evaluation of malaria. Such efforts are expected to improve the overall diagnosis and treatment of malaria, thereby contributing to a higher cure rate of malaria and a lower incidence of complications. The blood routine examination is a common test for malaria patients. During the acute attack, the WBC count and neutrophil count may increase, but they typically return to a normal range after the attack subsides. After repeated attacks, the WBC count decreases and the monocyte count increases. Additionally, the Hb and PLT will decrease to varying degrees. These indicators have been included in the guidelines for the diagnosis and treatment of malaria in China^5^. Notably, while there are numerous studies on thrombocytopenia in malaria, many of these studies have been conducted in regions with a high incidence of malaria, such as Africa^9,10^. In addition, there is a scarcity of research on PLT-based efficacy monitoring and prognosis evaluation in patients with malaria. Hence, a retrospective analysis was performed in the present study to investigate the correlation between PLT and Plasmodium density in patients with malaria. Investigating the value of PLT and associated parameters in predicting disease progression in malaria patients.

## Materials and methods

### Samples

**Figure 1:**
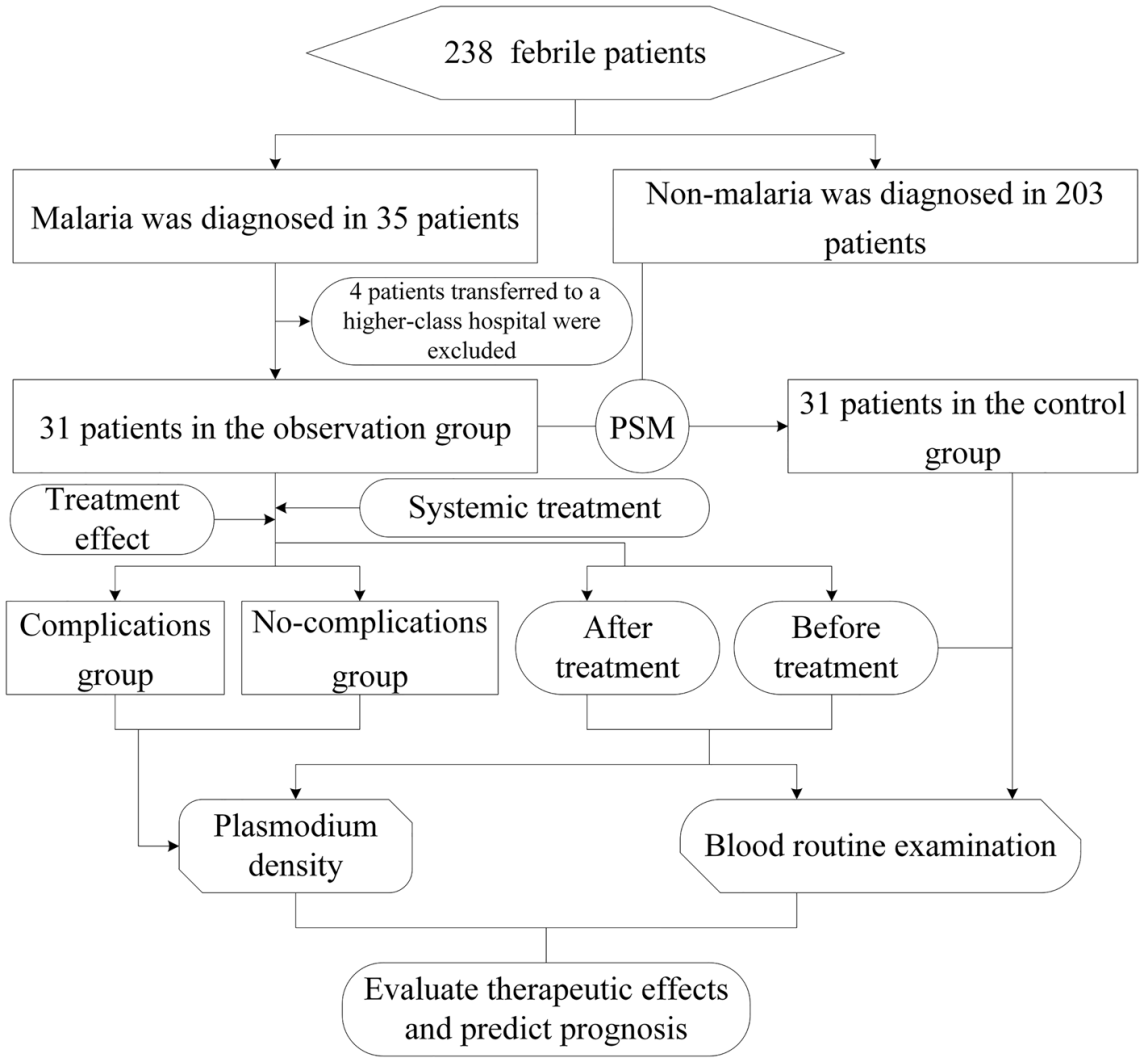
The recruitment process and study flow of participants.

In the present study, a total of 238 patients who were admitted to the hospital due to a fever from January 2018 to December 2022 and had participated in physical examinations within past one year were selected and retrospectively analyzed. The Platelet Count (PLT) obtained during the physical examination (referred to as “baseline PLT”) serves as a key parameter for Propensity Score Matching (PSM). Subsequently, 31 patients with imported malaria were included in the observation group. In contrast, 31 non-malaria patients with a fever were selected by means of the propensity score matching (PSM) method (age, gender, and PLT in physical examinations) as the control group. The observation group consisted of 28 males and 3 females with a median age of 46 years (ranging from 24 to 57 years) and a baseline PLT of 185 (150–238) × 10^9^/L. The control group consisted of 27 males and 4 females, with a median age of 43 years (ranging from 24 to 57 years) and a baseline PLT of 182 (152–228) × 10^9^/L. There were no notable differences in the baseline data between both groups (*P* > 0.05). All patients were diagnosed based on positive laboratory etiological examination results and their history of living in malaria-endemic areas outside China before the onset of the disease (refer to the *Guidelines for Malaria Diagnosis and Treatment*)^5^. Exclusion criteria: ①Past history of malaria. ②Patients with underlying medical conditions, such as thalassemia, iron deficiency anemia, hypertension, diabetes, and liver diseases. When the patient was initially examined by a doctor (referred to as before treatment or the initial visit), no treatment was administered. Following the initial visit, venous blood samples were collected from the patient every other day for Plasmodium smear microscopy, colloidal gold standard method, and blood routine tests until consecutive negative smear results were obtained. Additionally, follow-up phone calls were made to the patients every 3 months for a duration of 2 years”. If the patient’s condition improved, with their body temperature and PLT levels returning to normal, and if Plasmodium blood smear detection yielded negative results on two occasions, they were considered clinically cured^11^ (hereinafter referred to as after treatment). In the present study, 31 patients met these criteria for clinical cure on day 5 of hospitalization. Notably, one patient with *Plasmodium ovale* experienced a relapse after 12 months (First visit in February 2022, recurrence in February 2023). The research process is shown in Fig. 1.

### Instruments and consumables

Plasmodium test reagents (colloidal gold method), which (vivax malaria, falciparum malaria, ovale malaria and subtertian malaria can all yield positive test results, while subtertian malaria can be tentatively identified or ruled out. However, distinguishing between the other three types of Plasmodium is challenging) was used to detect plasmodium, and was purchased from Guangzhou Wondfo Biotech Co., Ltd. (Guangzhou, China). The microscopic examination of blood smears was completed using the OLYMPUS BX43 microscope (Olympus, Japan) after staining with Wright-Giemsa staining solution from Zhuhai Beisuo Biological Technology Co., Ltd. (Zhuhai, China). The Sysmex XN 2000 Automatic Hematology Analyzer (Sysmex Corporation, Kobe, Japan) was used for routine blood examinations.

### Methods

#### Plasmodium was detected by light microscopy

Firstly, an appropriate amount of whole blood was taken on the same slide to prepare a blood film with a moderate thickness. After the blood film dried naturally, the thin blood film was fixed with pure methanol at the analytical grade. Subsequently, distilled water was used for hemolysis in the thick blood film. The thin and thick blood films were then stained with the Giemsa staining solution and microscopically examined. The presence of Plasmodium could be verified by observing the thick blood film. If Plasmodium was detected, typing was carried out using the thin blood film. Plasmodium density was determined by counting the number of Plasmodium parasites observed when examining 200 WBCs under the microscope. The Plasmodium density (× 10^9^/L) was calculated using the formula: (Number of Plasmodium/200) × WBC count.

#### Plasmodium was detected by means of colloidal gold assay

Firstly, 5 μL of whole blood was placed in the sample hole. Subsequently, 4 drops of buffer solution were added to the buffer hole, and the mixture was incubated for 15 min to observe the results. The determination of positive results was made in accordance with the product instructions.

#### Blood routine examination

Firstly, an EDTA-K2 anticoagulant vacuum blood collection vessel was used to collect 2 mL of blood from patients. Subsequently, the Sysmex XN 2000 automatic hematology analyzer was used for blood routine examinations.

### Methods of treatment

Upon diagnosis, all patients were treated with an integrated anti-Plasmodium regimen to eliminate Plasmodium gametophytes. Based on nutritional support, hepatoprotective treatment, and anti-infection, artesunate and dihydroartemisinin-piperaquine tablets were prescribed in combination with pyronaridine phosphate and primaquine phosphate to eradicate Plasmodium and reduce the recurrence rate. During the course of treatment, 7 patients displayed varying degrees of abnormal liver function indicators. Among these, 1 patient experienced a relapse 12 months after discharge and required readmission, while 2 patients exhibited varying degrees of abnormal renal function indicators. However, there were no significant alterations in the liver, kidney, lung, or heart indicators for the remaining 22 patients. Patients in the observation group were further categorized into the complication group and the non-complication group based on the presence or absence of treatment-related complications.

### Observation indicators

The observation indicators comprised: (1) the epidemiological characteristics of malaria; (2) the levels of RBC, Hb, PLT, MPV, and PDW in patients with or without malaria; (3) the correlation between Plasmodium density and PLT in patients with malaria; and (4) the dynamic changes of PLT in patients with malaria during treatment.

### Statistical analysis

SPSS26.0 was used for data processing, and normality testing was used to analyze the normally distributed data. The data were expressed as ‾x ± s. For comparison of the two groups, t-tests were used. The non-normally distributed data were expressed as median (M) [quartile (P_25_-P_75_)]. The nonparametric Mann-Whitney U test was used to compare the two groups with a significance level set at *P* < 0.05 to indicate statistical significance.

### Ethics approval and consent to participate

This study was conducted with approval from the Ethics Committee of First People’s.

Hospital of Linping District, Hangzhou. This study was conducted in accordance with the declaration of Helsinki. Written informed consent was obtained from all participants.

## Results

### Epidemiological characteristics and analysis of 31 malaria patients, microscopic examination, and colloidal gold assay results

**Figure 2:**
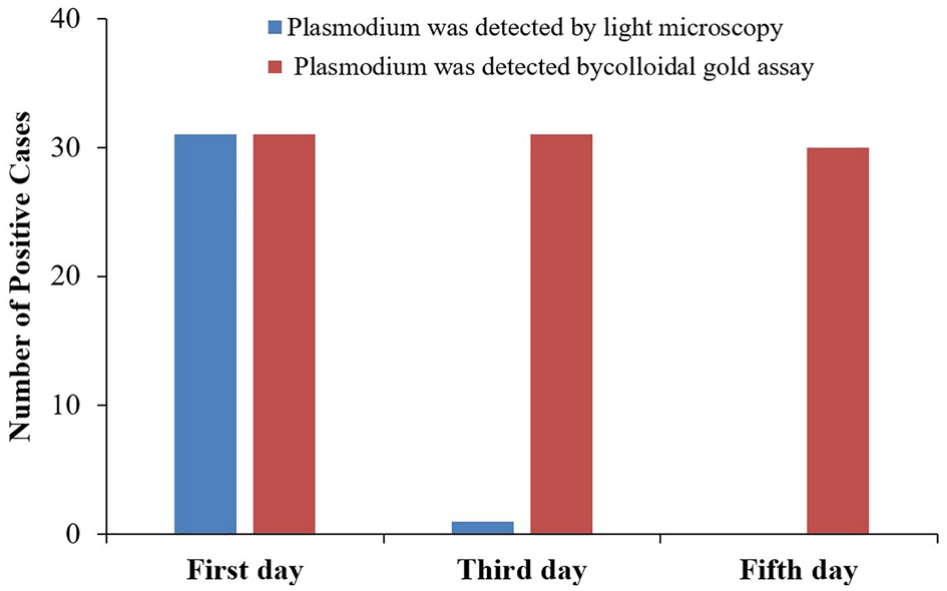
Microscopic examination and colloidal gold assay results.

In the present study, it was observed that the 31 malaria cases occurred sporadically throughout the year, with no distinct seasonal patterns. Among them, there were 28 patients with falciparum malaria (90.32%), 2 patients with vivax malaria (6.45%) and 1 patient with ovale malaria (3.23%). After 3 days of treatment, only 1 patient exhibited a positive result in microscopic examinations, and 31 patients exhibited positive results in the colloidal gold assay. After 5 days of treatment, all patients presented negative microscopic examination results, while 30 patients presented positive colloidal gold assay results (Fig. 2).

### Comparison of blood routine parameters between the observation group and the control group

The WBC, RBC, Hb, and PLT of patients in the observation group were lower than those in the control group. Among major blood routine parameters, there was only a significant difference in PLT between the control group and the observation group (*P* < 0.05). The MPV and PDW in the observation group were lower than those in the control group, but the difference was not statistically significant (*P* > 0.05), as shown in Table 1.

**Table 1:** Blood routine parameters of patients.

Item	Observation group	Control group	Statistical value	P value
Cases (n)	31	31	–	–
WBC(× 10^9/^L)	5.2(4.5 ~ 6.5)	5.8(4.0 ~ 8.7)	Z = − 0.725	0.468
RBC(× 10^12/^L)	4.689 ± 0.779	4.821 ± 0.513	t = − 0.784	0.436
Hb(g/L)	139.97 ± 21.897	144.355 ± 16.520	t = − 0.890	0.377
PLT(× 10^9/^L)	84(53 ~ 132)	190(152 ~ 245)	Z = − 5.611	< 0.001
MPV(fL)	10.387 ± 1.202	10.490 ± 1.362	t = − 0.316	0.753
PDW(%)	13.600 ± 2.441	13.603 ± 2.866	t = 1.474	0.146

The blood routine results are the first measurement results at the time of the onset of the disease.

*WBC* white blood cells count, *RBC* red blood cells count, *Hb* hemoglobin, *PLT* platelet count, *MPV* mean platelet volume, *PDW* platelet distribution width.

### Correlation analysis between PLT and Plasmodium density

**Figure 3:**
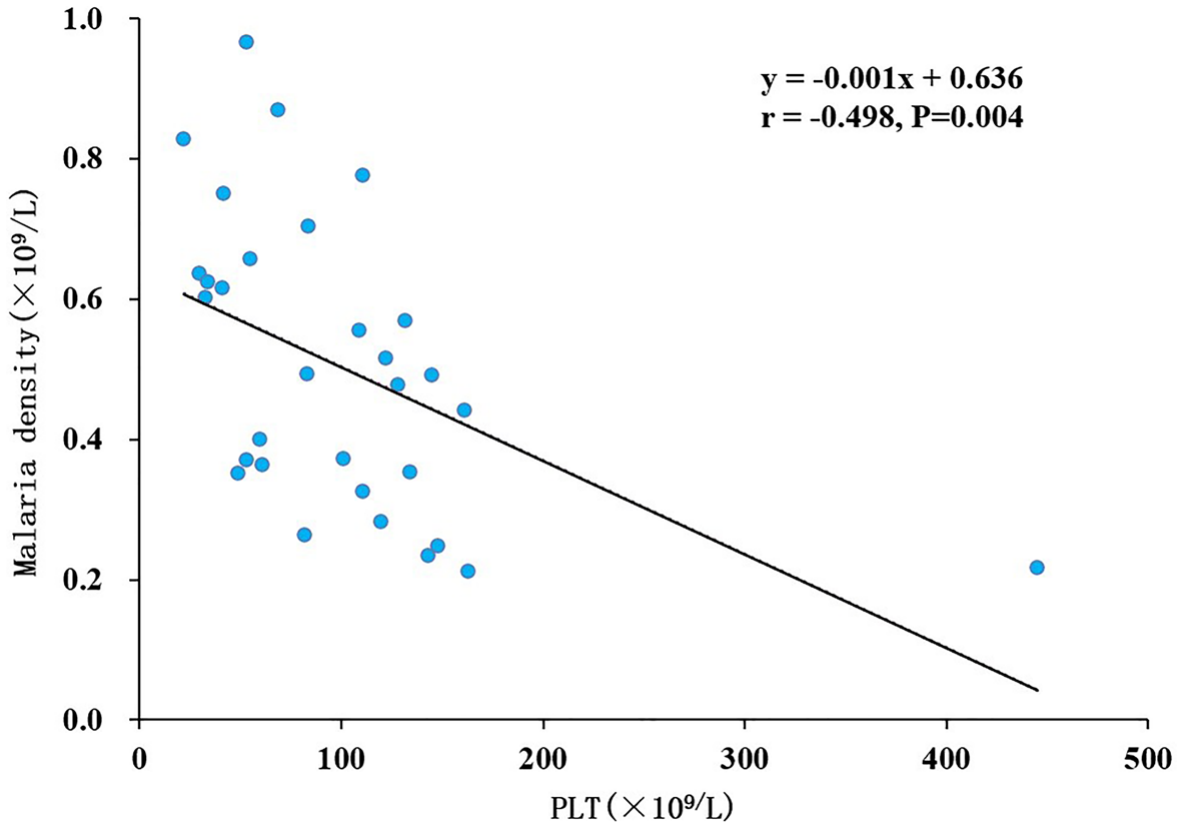
Correlation between PLT and Plasmodium density in patients with malaria.

A total of 22 individuals diagnosed with malaria exhibited thrombocytopenia (< 125 × 10^9^/L), constituting 70.97% of the observed cases. The results of statistical analysis based on correlation coefficients reveal that there was a negative correlation between PLT and Plasmodium density. However, the correlation is weak as r is close to 0.5 (Fig. 3).

### Changes in blood routine parameters in the observation group before and after treatment

The WBC, RBC, Hb, PLT, MPV, and PDW levels of patients in the observation group after treatment were higher than those before treatment. There were no significant differences in the WBC, RBC, and Hb levels before and after treatment (*P* > 0.05). The differences in PLT, MPV, and PDW levels before and after treatment were statistically significant (*P* < 0.05), as shown in Table 2.

**Table 2:** Blood routine parameters of patients with malaria before and after treatment.

Item	Before treatment	After treatment	Statistical value	P value
Cases (n)	31	31	–	–
WBC(× 10^9/^L)	5.2(4.5 ~ 6.5)	6.2(5.5 ~ 7.8)	Z = − 0.725	0.468
RBC(× 10^12/^L)	4.689 ± 0.779	4.697 ± 0.804	t = − 0.035	0.972
Hb(g/L)	139.97 ± 21.897	140.645 ± 22.743	t = − 0.119	0.905
PLT(× 10^9/^L)	84(53 ~ 132)	146(102 ~ 198)	Z = − 3.682	< 0.001
MPV(fL)	10.387 ± 1.202	12.897 ± 1.573	t = − 7.060	< 0.001
PDW(%)	13.600 ± 2.441	17.819 ± 2.637	t = − 6.538	< 0.001

*WBC* white blood cells count, *RBC* red blood cells count, *Hb* hemoglobin, *PLT* platelet count, *MPV* mean platelet volume, *PDW* platelet distribution width.

### Comparison of PLT and associated parameters between the complication group and the non-complication group

According to the occurrence of complications during treatment, patients in the observation group were subdivided into a complication group and a non-complication group. Before treatment, there were no significant differences in MPV and PDW levels between the complication group and the non-complication group (*P* > 0.05), but there was a significant difference in PLT and malaria density between the two groups. After treatment, the PLT of patients in the complication group was lower than that in the non-complication group, and the MPV and PDW levels of patients in the complication group were higher than those in the non-complication group. There were significant differences in PLT and PDW levels between the complication group and the non-complication group (*P* < 0.05), but no significant difference in MPV between the two groups (*P* > 0.05). The PLT, MPV, and PDW levels of patients in the two groups after treatment were significantly higher than those before treatment (*P* < 0.05), as shown in Table 3.

**Table 3:** Changes in PLT, MPV, PDW and Malaria density in the complication group and the non-complication group before and after treatment.

Item	Before treatment				After treatment			
	Complication group	Non-complication group	Statistical value	P value	Complication group	Non-complication group	Statistical value	P value
Cases (n)	9	22	–	–	9	22	–	–
PLT(× 10^9/^L)	49(33 ~ 68)^a^	115(67 ~ 143)^d^	Z = − 3.069	0.002	89(73 ~ 115)^a^	171(132 ~ 226)^d^	Z = − 3.330	0.001
MPV(fL)	10.633 ± 1.068^b^	10.286 ± 1.262^e^	t = 0.724	0.475	13.300 ± 1.456^b^	12.732 ± 1.621^e^	t = 0.746	0.462
PDW(%)	13.856 ± 2.600^c^	13.496 ± 2.428f.	t = 0.367	0.727	19.456 ± 1.691^c^	17.150 ± 2.689f.	t = 2.907	0.009
Malaria density(× 10^9^/L)	0.619 ± 0.198	0.454 ± 0.192	t = 2.134	0.041	–	–	–	–

*PLT* platelet count, *MPV* mean platelet volume, *PDW* platelet distribution width.

^a^Z =  − 2.696, P = 0.007.

^b^t =  − 4.431, P < 0.001.

^c^t =  − 5.416, P < 0.001.

^d^Z =  − 5.683, P < 0.001.

^e^t =  − 5.584, P < 0.001.

^f^t =  − 4.731, P < 0.001.

### Dynamic changes in the PLT, MPV, and PDW of patients with malaria during treatment

**Figure 4:**
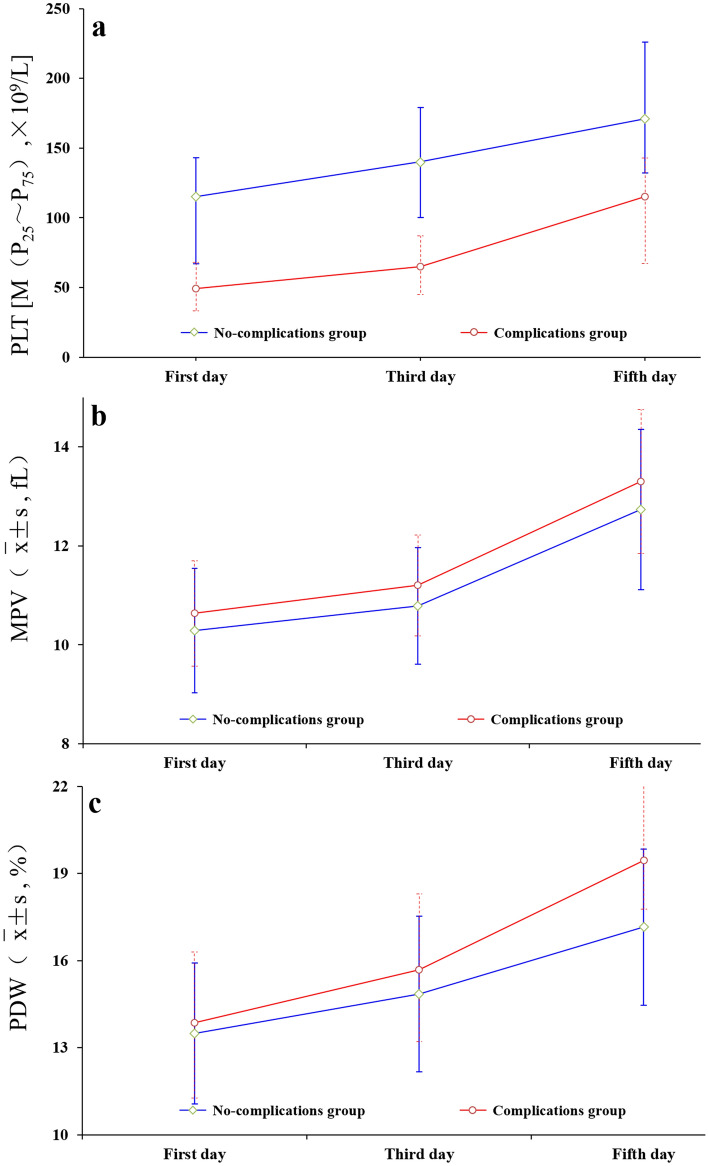
Dynamic changes in PLT, MPV and PDW levels in both groups within 5 days of admission (Note: a: PLT; b: MPV; c: PDW).

The PLT, MPV, and PDW levels in the complication group and the non-complication group increased gradually from the time of admission to the 3rd and 5th days of treatment. The PLT in the complication group was consistently lower than that in the non-complication group, while the MPV and PDW levels in the complication group were higher than those in the non-complication group, as shown in Fig. 4.

## Discussion

In the present study, findings were made that the WBC, RBC, and Hb of patients with malaria were lower than those of non-malaria patients with a fever, but the differences were not statistically significant. Ali E^10^ and ManasKotepui^12^ demonstrated that there were statistically significant differences in WBC, RBC, and Hb between patients with malaria and those without malaria. Notably, the results of the present study do not wholly align with their research results. The PLT of patients with malaria was significantly lower than that of non-malaria patients with a fever, but there were no significant differences in MPV and PDW levels between the two groups. Again, the results are not completely consistent with those of Ali E^10^. Such disparity could potentially be attributed to the patients included in the present study suffering from imported malaria and having a defined epidemic history. Additionally, the majority of patients sought medical advice immediately after the onset of malaria and received malaria screening in time, which contributed to early diagnosis. Akin to Ali E^10^ and ManasKotepui^12^, the research results of Li Hui Xia^13^, a Chinese scholar, are also not completely consistent with those of the present study. The possible reason may be that Li Hui Xia’s research results were compiled before 2016, a period when malaria had not yet been eliminated in China. In parallel, patients in the present study suffered from imported malaria, and the differences between the two research populations induced inconsistent results. The present results reveal that there was a negative correlation between Plasmodium density and PLT, which is consistent with the findings of Kochar DK et al.^14^. As such, PLT was demonstrated to be closely related to the severity of malaria, thereby laying a foundation for further exploring the value of PLT and associated parameters in efficacy evaluation in patients with malaria.

Although PLT decreased significantly in patients with malaria, there were many factors causing the decline. The rapid advancement of detection technologies has provided clinical practice with the benefits of convenient, precise, and efficient detection methods, which significantly aid in the screening and supporting diagnosis of malaria patients^15,16^. Hence, the diagnostic value of PLT and associated parameters for malaria was not deeply explored in the present study. Presently, it is not feasible to substitute PLT counts for Plasmodium microscopic examinations and rapid detection methods in the diagnosis of malaria. Nevertheless, Plasmodium microscopic examinations and rapid detection methods have their limitations in terms of effectively monitoring treatment outcomes and assessing the prognosis of malaria patients. Therefore, the changes in PLT and associated parameters in the treatment of patients with malaria were comprehensively investigated to identify the significance of PLT and associated parameters in efficacy monitoring and prognosis evaluation of patients with malaria.

In the present study, it was demonstrated that the WBC, RBC, Hb, PLT, MPV, and PDW levels of patients in the observation group after treatment were higher than those before treatment. Further, the differences in PLT, MPV, and PDW levels before and after treatment were statistically significant. Such results are basically consistent with those of Guo Wen Juan et al.^17^. Thus, PLT-related parameters can be used to evaluate the efficacy in patients with imported malaria. In clinical practice, it has been observed that most patients with non-severe malaria tend to show negative results in microscopic examinations within 1–2 days after receiving standard treatment. Conversely, positive results from rapid detection (colloidal gold assay) may persist for 1–2 weeks after standard treatment, which aligns with the findings of Dalrymple U et al.^18^. Additionally, PLT levels tend to gradually increase during this period. In the present study, PLT and associated parameters were further analyzed in patients with malaria in the complication group and the non-complication group. The results indicate that the PLT, MPV, and PDW levels in the complication group and the non-complication group exhibited an upward trend after treatment. Additionally, the PLT of patients in the complication group was significantly lower than that in the non-complication group, while the MPV and PDW levels of patients in the complication group were higher than those in the non-complication group. This approach can help reduce complications and mitigate the risk of an unfavorable prognosis. Moreover, it is also required to monitor the changes in PLT, MPV, and PDW in patients with malaria during treatment. The results indicate that standard treatment could not only eradicate Plasmodium quickly, but also gradually restore PLT to a baseline range. Accordingly, MPV and PDW gradually increased. At the same time, PLT, MPV, and PDW in patients with or without complications exhibited a consistent change trend during treatment. However, PLT in the complication group was significantly lower than that in the non-complication group, while MPV and PDW in the complication group were higher than those in the non-complication group. Such findings can be ascribed to the platelets being activated after Plasmodium infection, and the secretion of acute reactive proteins and the activation of platelets being enhanced in the liver through cytokines such as IL-1β and TGF-β, thereby eliminating Plasmodium in the body. In addition, activated platelets can also activate endothelial cells and leukocytes, thereby promoting a series of inflammatory reactions in vivo. Platelet consumption in this process may be the main contributor to platelet reduction caused by Plasmodium infection.

As platelet levels decrease in the body, new platelets may be produced, leading to an increase in MPV and PDW^19–21^. These findings emphasize the importance of continuous monitoring of PLT and related parameters in malaria patients throughout the course of treatment. Such measures can not only contribute to effective efficacy monitoring, but are also conducive to scientific prognosis evaluation and timely adjustment of therapeutic regimens, thereby promoting the rehabilitation of patients with malaria.

## Conclusion

PLT decreases significantly in patients with imported malaria. Following effective treatment, PLT levels gradually return to their baseline range. Therefore, it is crucial to closely monitor PLT levels and related parameters in order to identify patients with higher parasite density, those at risk of developing complications, and those who may test negative for the parasite on a blood smear. When PLT approaches the clinical critical value, the changes in patients should be closely observed.

## Data Availability

The datasets used and/or analysed during the current study available from the corresponding author on reasonable request. We declared that materials described in the manuscript, including all relevant raw data, will be freely available to any scientist wishing to use them for non-commercial purposes, without breaching participant confidentiality.
